# One Ring to Contain Them all: The Pivotal Role of the Built Environment in all Pillars of Lifestyle Medicine

**DOI:** 10.1177/15598276251329818

**Published:** 2025-03-25

**Authors:** Levi Frehlich, Jonathan Bonnet

**Affiliations:** 1 Cumming School of Medicine, 70401 University of Calgary, Calgary AB, Canada (LF); 2 Stanford Lifestyle Medicine, 6429 Stanford University School of Medicine, Palo Alto, CA, USA (LF, JB)

**Keywords:** Wellness, healthy living, population health, public health, urban planning

## Abstract

The built environment – defined as the human-made physical aspects of where people live, work, and play – has long influenced morbidity and mortality. While historical examples include environmental exposures such as climate extremes or access to clean water, modern urbanization presents distinct health challenges and opportunities. This article explores the built environment’s role in shaping health through the lens of lifestyle medicine, encompassing six key pillars: nutrition, physical activity, stress management, sleep health, social connection, and avoidance of risky substances. Neighbourhood food environments affect dietary behaviours, with greater access to fast food linked to obesity and cardiovascular disease, while fresh food availability promotes healthier choices. Walkability and greenspace enhance physical activity, while urban design incorporating green- and blue-spaces supports stress management. Environmental noise and artificial light at night impact sleep quality, whereas community infrastructure fosters social connectedness. Lastly, the spatial distribution of alcohol and tobacco outlets influences substance use behaviours. Given the built environment’s wide-ranging influence, designing neighbourhoods that naturally promote health could yield significant public health benefits. This perspective underscores the need for policy-driven urban planning that prioritizes health-supportive environments, making the healthy choice the default choice for populations.

‘A recent systematic review found that forest bathing can significantly reduce symptoms of depression and anxiety’.

## What is the Built Environment?

Where you live has had an influence on the risk for morbidity and mortality since the dawn of time. Historically, environmental examples could involve living by the water and risk of flood or in climates that expose oneself to extreme heat or cold. However, as societies started to settle and densify, different risks for morbidity and mortality became apparent.

One well cited historical example is the cholera outbreak in the 19^th^ century. Dr. John Snow noticed that individuals that consumed water from a specific location (the Broad Street pump) were more likely to develop cholera than individuals consuming water elsewhere.^1^ Although not the first, this example illustrates the importance of the built environment in shaping health.

The built environment can be broadly classified as the human-made physical aspects of the environment in which we live, work, and play.^2,3^ The built environment fits prominently in the socio-ecological model of health, which posits that health is a function of many overlapping influences including individual, interpersonal, organizational, community, and policy or micro, meso, and macro environmental factors.^4^ Although the built environment fits prominently under community influence, it could be argued that its connection with urban planning and city policy could also bring the built environment to the policy level or meso-macro link. A recent article describing how lifestyle medicine can influence cardiometabolic diseases used this framework.^5^ Therefore, it could be argued that the built environment has the ability to influence all six areas of lifestyle medicine (nutrition, sleep health, social connection, physical activity, stress management, and substance use) – ‘one ring to rule them all’.

This article explores how the built environment integrates into all of the pillars. While multiple systematic reviews have been completed on each topic,^6-20^ this article seeks to frame these built environment findings within a lifestyle medicine context.

## The Built Environment and Nutrition

The built environment can influence healthy dietary patterns by varying what food choices are available to its residents, a term commonly referred to as the ‘neighbourhood food environment’. Two recent systematic reviews aimed to elucidate the impact of the neighbourhood food environment on obesity metrics.^6,7^ Results of one of these reviews using Chinese literature found 17 studies, of which 11 reported on the relationship the neighbourhood food environment and weight status.^7^ Within the 11 reported studies there were 10 reporting statistically significant associations and one reporting null associations.^7^ Significant associations were between western style fast-food outlets and markers of obesity (increased body mass index and waist-to-hip ratio). For example, one study indicated that each additional fast-food restaurant opening in the past was associated with a future increase in waist-to-hip ratio of 0.46 and 0.38 increases for rural women and men, respectively; however, it was also associated with a 0.53 future decrease in waist-to-hip ratio in urban women.^21^ Further, another study found that the density of fresh food markets was associated with a lower overall incidence of BMI measured obesity (standardized linear beta −0.598).^22^

The second review was restricted to randomized controlled trials (RCTs) or analyses using difference-in-difference methods, or instrumental variable analysis.^6^ Fifty-eight RCTs studies were included, with 55 being from high-income countries. Results varied with location and sociodemographic characteristics, for example, the authors found that non-white rural adult populations from the United States self-reported more fast-food intake when fast-food restaurants were closer or more densely populated in their neighbourhood,^6^ although results were null for white populations.^6^ Another systematic review assessed the association between the neighbourhood food environment and cardiovascular disease found that higher access to fast-food was associated with higher cardiovascular morbidity and mortality.^8^ For example, among longitudinal studies one found a 10% increase in fast-food restaurant density was associated with a higher relative risk (1.39, 95%CI: 1.19 to 1.63) of cardiovascular mortality over four years.^23^ While preliminary results are encouraging finding a general theme that suggested increasing fresh food and decreasing fast-food may promote better health, findings perhaps unsurprising to this readership, more research is needed across diverse populations and locations.

## The Built Environment and Physical Activity

Mounting evidence has shown that neighbourhood ‘walkability’ is positively associated with levels of physical activity, and in particular active transportation.^9^ A recent umbrella review on the associations between the built environment and physical activity in adults found 116 individual systematic reviews on the topic.^9^ Although most evidence was observational, there was consistent evidence, with moderate-to-high certainty, that walkability supports transportation physical activity. Moreover, there was moderate certainty of evidence that neighbourhood walkability and greenspace increases overall physical activity.^9^ Walkable neighbourhood environments can also support physical activity interventions.^10^ For example, in a systematic review on the associations between the built environment and intervention-facilitated physical activity, the authors found the majority of identified studies showed either an amplification of the intervention or no influence.^10^ The totality of the evidence surrounding the built environment and physical activity shows promise for increasing population levels of physical activity.

## The Built Environment and Stress Management

Urban planners and city designers have the opportunity to incorporate green- and blue-space into neighbourhoods, as doing so may aid with stress management.^11-13^ A systematic review on urban green and blue infrastructure found that out of 19 experimental studies identified, 15 reported positive psychological and physical influences on stress.^11^ Further, another systematic review on the neurobiological effects of the built and natural environment on mental health found that natural settings were linked to greater alpha brain wave activity measured by electroencephalography which is an indicator of relaxation and restoration.^12^ These results align with the practice of forest bathing which involves mindfully immersing oneself in forested areas. A recent systematic review found that forest bathing can significantly reduce symptoms of depression and anxiety (standardized mean difference compared to control −0.67 95%CI: −0.99 to −0.35 and −0.84 95%CI: −1.42 to −0.25, respectively).^13^

## The Built Environment and Restorative Sleep

In 2018 the World Health Organization published a report linking environmental noise to human health, including sleep.^18^ A 2022 systematic review and meta-analysis updated these findings including 36 studies on the association between environmental noise and sleep.^14^ Exposure to aircraft, road, and railway noise, led to significant increases in sleep disturbance (odds ratio 2.30 [95%CI: 1.87 to 2.82], 1.80 [95%CI: 1.50 to 2.17], and 2.14 [95%CI: 1.54 to 2.97], respectively).^14^ Moreover, the association between environmental noise and sleep disturbance appeared to indicate a positive dose-response relationship with increasing decibel levels.^14^ Although relatively less studied, another source of built environment exposure that may disrupt sleep is artificial light at night. Another systematic review looking at neighbourhoods and sleep health found one study indicating that highly artificially lit neighbourhood areas at night were associated with increased odds of short sleep (OR 1.16 [95%CI: 1.10 to 1.22] for women, and 1.25 [95%CI: 1.19 to 1.31] for men).^19,24^

## The Built Environment and Social Connection

An updated 2023 systematic review of the built environments ability to support social connection found 51 articles on the topic.^15^ The authors conclude that there is strong evidence that community hubs, neighbourhood design, and green- and blue-space have positive impact on social connection.^15^ For example, ‘Play Streets’ in Australia streets where streets were closed to vehicle traffic and allowed pedestrian use, resulted in 91.7% of respondents saying they felt an increased sense of belonging, and 95% of respondents indicated they got to know more people.^15^ Moreover, a 2015 meta-analysis found significant associations between neighbourhood walkability (effect size = 0.20, 95% CI = [0.15, 0.26]), and land-use mix (effect size = 0.29, 95% CI = [0.16, 0.42]), on community participation in older adults.^20^

## The Built Environment and Avoidance of Risky Substances

The similarities between the built environment and avoidance of risky behaviours parallels that of the food environment and comes down to ease of access. A 2022 systematic review and meta-analysis on the associations of tobacco retailer density and proximity with tobacco use in adults found that lower density and farther proximity lead to statistically significant relative risk reduction of 2.06% (95% CI: 1.26 to 2.85) in smoking and 6.50% (95% CI: 4.64 to 8.37) increase in quitting smoking.^16^ Moreover, a similar relationship is found with alcohol consumption where another 2022 systematic review on alcohol consumption patterns found that proximity to alcohol outlets was one of the main factors driving consumption albeit in nuanced ways based on geography with the authors stating ‘For instance, in Europe, the association with increased outlets moderates the heritability of alcohol [problems]; in Australia, it was related to the mean daily intake; and in North America, it was found to be related to high consumption’.^17^

## Food for Thought

Data have convincingly shown that ZIP code can matter more to health than Genetic Code.^25,26^ ZIP codes also consciously and subconsciously influence lifestyle behaviours, which have been shown to prevent 80% of chronic disease^27^ and 40% of premature mortality.^28^ Imagine counselling a patient to make improvements in their lifestyle and they go home to one of two environments. The first is a neighbourhood with an abundance of fast-food, no sidewalks, little to no greenspace, poorly built housing that allows in light and noise, no places to meet up with friends, and convenience stores with tobacco and alcohol products. The second is a neighbourhood with fresh farmers market food, well maintained sidewalks, green- and blue-space, housing built to keep light and noise to a minimum, many retail destinations to meet up with friends, and limited access to tobacco and alcohol products. Clearly these examples are stark, and there are many factors that influence each individual area. However, as Dr. David Katz has stated ‘the choices people make are subordinate to the choices they have’.

Moreover, we know that although addressing one pillar of lifestyle medicine is beneficial, addressing multiple or all of them have synergistic effects,^29^ the same could be true for modifications of the built environment. The supportiveness of a health supporting neighbourhood may seem intuitive, and have been the focus of multiple government reports, including from the Chief Public Health Officer in Canada^2^ and the Surgeon General in the United States.^3^ Initiatives such as the Blue Zones Project have great potential to influence population health.^30^ The Blue Zones Project aimed to take the information learnt from long lived populations around the world and incorporate it into other communities. In one example the community of Albert Lea, Minnesota created more walkable streets and provided greater access to healthy food options, the result was a 40% decrease in health care costs and a 3.2 year increase in life expectancy.^30^ Although the Blue Zone Projects consider many of the pillars mentioned above, there is opportunity for future interventions to simultaneously take all of the above aspects on the built environment into account. If the results of incorporating a few aspects of the built environment produce profound changes in health, incorporating all aspects has great untapped potential.

The incremental and compounding benefits of the built environment or difficult to accurately assess. As new research is conducted and the magnitude of benefits are solidified, it will be difficult to ignore the profound effect on health that built environments have. This does not diminish the importance of individual autonomy and personal lifestyle choices. These individual choices will ultimately determine the effect on health. However, the built environment can either facilitate and support these choices, or deter and detract from individuals being able to make them. In short, neighbourhoods can be constructed in a way that makes the healthy choice the default choice.
